# Fight evolution with evolution: plasmid-dependent phages with a wide host range prevent the spread of antibiotic resistance

**DOI:** 10.1111/eva.12076

**Published:** 2013-06-10

**Authors:** Ville Ojala, Jarkko Laitalainen, Matti Jalasvuori

**Affiliations:** 1 Centre of Excellence in Biological Interactions, Department of Biological and Environmental Science and Nanoscience Center, University of JyväskyläJyväskylä, Finland; 2 Division of Ecology, Evolution and Genetics, Research School of Biology, Australian National UniversityCanberra, ACT, Australia

**Keywords:** evolution of antibiotic resistance, conjugation, conjugative plasmid-dependent phages, phage therapy

## Abstract

The emergence of pathogenic bacteria resistant to multiple antibiotics is a serious worldwide public health concern. Whenever antibiotics are applied, the genes encoding for antibiotic resistance are selected for within bacterial populations. This has led to the prevalence of conjugative plasmids that carry resistance genes and can transfer themselves between diverse bacterial groups. In this study, we investigated whether it is feasible to attempt to prevent the spread of antibiotic resistances with a lytic bacteriophage, which can replicate in a wide range of gram-negative bacteria harbouring conjugative drug resistance–conferring plasmids. The counter-selection against the plasmid was shown to be effective, reducing the frequency of multidrug-resistant bacteria that formed via horizontal transfer by several orders of magnitude. This was true also in the presence of an antibiotic against which the plasmid provided resistance. Majority of the multiresistant bacteria subjected to phage selection also lost their conjugation capability. Overall this study suggests that, while we are obligated to maintain the selection for the spread of the drug resistances, the ‘fight evolution with evolution’ approach could help us even out the outcome to our favour.

## Introduction

The rapidly increasing number of antibiotic-resistant bacterial infections is of a major concern to modern health care worldwide, causing both substantial financial loss and numerous deaths (Taubes 2008; Bush et al. 2011). From an evolutionary standpoint, the problem is hardly a surprising one, because the constant application (both appropriate and inappropriate) of antibiotics has exerted a strong pressure on bacteria to develop resistance (Cohen 1992; Levin et al. 1997; Austin et al. 1999; Levy and Marshall 2007). Nevertheless, the predictability of the issue has not made it any easier to deal with, and in the case of many serious bacterial pathogens, such as methicillin-resistant *Staphylococcus aureus* (MRSA) or certain strains of *Pseudomonas aeruginosa* and *Klebsiella pneumoniae*, the number of viable treatment options is dangerously declining (MacKenzie et al. 1997; Livermore 2002; Taubes 2008; Wise et al. 2011). The situation is further complicated by the fact that only a few novel classes of antibiotics have been introduced during the past 50 years (Walsh 2003; Coates et al. 2011).

Resistant bacteria commonly harbour mobile genetic elements, such as conjugative plasmids that contain genes conferring resistance to several classes of antibiotics (Bennett 2008). Conjugative plasmids replicate independently of the host genome and they can facilitate their own transfer from one bacterial strain or species to another by coding for a channel through which a copy of the plasmid is transferred from donor to recipient cell (Brinton 1965). This horizontal gene transfer (HGT) allows for a highly efficient spread of resistances in bacterial communities (Davies 1994; Grohmann et al. 2003). Autonomous replication and the ability to move between (sometimes distantly related) bacteria mean that conjugative plasmids are independently evolving genetic elements (Norman et al. 2009). Consequently, their presence and horizontal movement can, depending on the circumstances, be an advantage, a disadvantage or neutral in terms of fitness of both the host they reside in and the other bacteria in the microbial community (Eberhard 1990; Kado 1998; Dionisio et al. 2005; Slater et al. 2008; Norman et al. 2009). For example, the transfer of a conjugative plasmid from a bacterial donor to a (unrelated) recipient could potentially lower the fitness of the donor and increase the fitness of the recipient if the two bacteria compete over resources, and the possession of the plasmid provides some competitive advantage, such as resistance to the antibiotics present in the system (Jalasvuori 2012).

Interfering with the process of bacterial conjugation has been proposed as one potential way of combating the spread of plasmid-mediated antibiotic resistances (Smith and Romesberg 2007; Williams and Hergenrother 2008). Certain bacteriophages (phages) specifically infect and kill conjugative plasmid–harbouring bacteria (Caro and Schnös 1966). These phages use conjugative plasmid–encoded proteins as their receptor to gain entrance to a host cell. The host range of a given conjugative plasmid–dependent (or male-specific) phage is therefore mainly determined by the host range of suitable conjugative plasmids (Olsen et al. 1974). In practice, conjugative plasmid–dependent phages are natural enemies of both the conjugative plasmids and the bacteria that harbour them. A previous study suggests that in the absence of antibiotic selection and other bacteria, the presence of a lytic conjugative plasmid–dependent phage can efficiently select for bacteria that either have lost their conjugative plasmids or harbour a conjugation-deficient version of the plasmid (Jalasvuori et al. 2011). However, the capability of these phages to limit the rate of horizontal transfer of plasmids between bacteria was not investigated. Other studies have shown that nonlytic filamentous phages are capable of preventing the spread of conjugative plasmids by physically inhibiting conjugation (Novotny et al. 1968; Lin et al. 2011).

**Figure 1:**
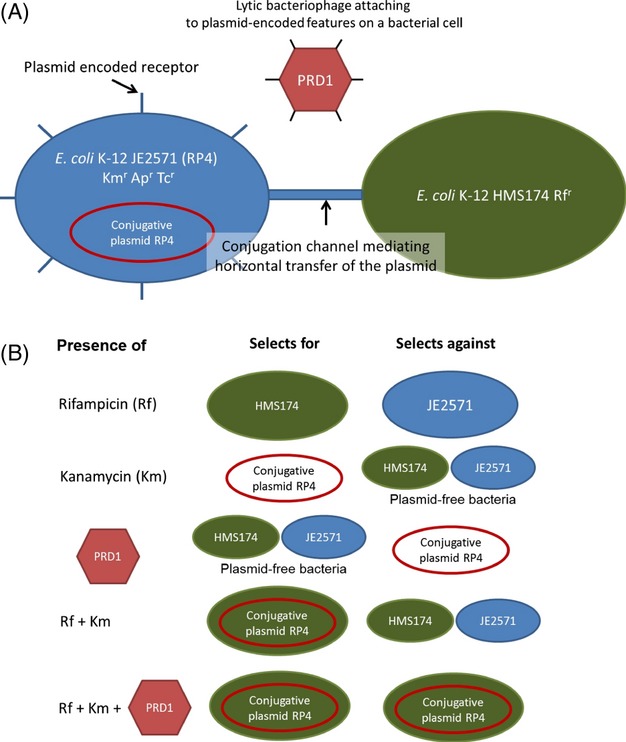
Schematic presentation of the experimental setup and the selection pressures.

Elaborating from these previous studies, we here investigated whether a lytic conjugative plasmid–dependent phage can prevent the emergence of new multiresistant strains by selecting against the plasmid or, more specifically, the plasmid-encoded sex apparatus facilitating the transfer of the plasmid to other bacteria. Moreover, we measure how much the presence of nonlethal antibiotic selection favouring different plasmid and bacterium combinations alters the counter-selective effect of phages (Fig. 1). In our experiments, two antibiotic-resistant bacterial strains of *Escherichia coli* K-12 were cultivated in daily replenished cultures together for 3 days. One of the used strains, JE2571(RP4), contains a conjugative plasmid RP4 conferring resistance to several antibiotics of different classes (ampicillin, kanamycin and tetracycline), whereas the other strain HMS174 is plasmid free but resistant to rifampicin due to a chromosomal mutation. In this experimental setup, the potential conjugative transfer of the RP4 plasmid from JE2571(RP4) to HMS174 would create a new multiresistant strain HMS174(RP4). The presence of the conjugative plasmid–dependent phage PRD1 selects against all bacteria representing plasmid-encoded receptors on the cell surface. Bacteria are resistant to phage infections if they are free of the plasmid or they harbour a conjugation-defective mutant (Jalasvuori et al. 2011).

Altogether, we here demonstrate that conjugative plasmid–dependent phage PRD1 effectively restricts the emergence of the multiresistant HMS174(RP4) strain even in the presence of nonlethal antibiotic selection. While growth-reducing antibiotic concentrations may play an important role in the evolution of bacterial antibiotic resistance (Andersson and Hughes 2012), these results suggest that is possible to combat this evolution with counter-selective attempts.

## Materials and methods

### Bacterial strains, bacteriophages and culture conditions

*Escherichia coli* K-12 strains JE2571(RP4) (Bradley 1980), HMS174 (Campbell et al. 1978) and JM109(pSU19) were used in this study. JE2571 harbours a conjugative incompatibility group P plasmid RP4 (Datta et al. 1971), which induces antibiotic resistance to kanamycin, ampicillin and tetracycline. HMS174 contains chromosomal rifampicin resistance. JM109(pSU19) contains a nonconjugative plasmid pSU19 (Bartolomé et al. 1991) that induces chloramphenicol resistance. All strains were cultivated in Luria–Bertani (LB) medium (Sambrook et al. 1989) at 37°C. Shaking at 200 revolutions per minute (rpm) was used, with the exception of the evolution experiments where the cultures were unshaken. For general antibiotic selection, kanamycin, rifampicin and chloramphenicol were used in final concentrations of 32 μg/mL, 55 μg/mL and 25 μg/mL, respectively. The bacteriophage used in this study was PRD1; a lytic conjugative plasmid–dependent phage infecting a wide range of gram-negative bacteria that contain conjugative plasmids belonging to incompatibility groups P, N and W (Olsen et al. 1974).

### Evolution experiments

5 μL of JE2571(RP4) and HMS174 overnight cultures were inoculated into the same tube containing 5 mL of fresh LB medium. The mixed cultures were treated with (i) no antibiotics, (ii) kanamycin, (iii) rifampicin or (iv) kanamycin and rifampicin. When appropriate, kanamycin and rifampicin were added in nonlethal but growth-reducing concentrations of 3.2 μg/mL and 3.7 μg/mL, respectively (Fig S1A,B). Each antibiotic treatment was performed both in the presence and in the absence of conjugative plasmid–dependent phage PRD1. Immediately after the transfer of the bacteria, 5 μL of phage stock containing approximately 10^11^ plaque-forming units per millilitre (pfu/mL) was added to the appropriate treatments. Cultures were grown at 37°C without shaking. The length of the experiment was approximately 72 hours, and the cultures were renewed at 24- and 48-hour time points by transferring 5 μL of culture to 5 ml of fresh LB medium (containing the appropriate antibiotics; no new phage was added during the refreshments). Each treatment was sampled during the culture renewals and at the end of the experiment. These samples were diluted and plated on either regular or antibiotic-containing (kanamycin and rifampicin) 1% LB agar plates to obtain the total bacterial densities and the number of bacteria resistant to both antibiotics. Also, from all treatments, a random sample of clones (n_total_ = 210) growing on kanamycin- and rifampicin-containing plates were transferred to kanamycin-, tetracycline-, ampicillin- and rifampicin-containing plates to further confirm that the formed multiresistant clones harbour a plasmid (i.e. controlling the frequency of spontaneous antibiotic-resistant mutants). In addition, final phage densities were determined at the end of the experiment by plating diluted samples (on 1% LB agar plates with a 0.7% soft agar overlay) from phage-containing treatments with the ancestral form of JE2571(RP4) bacteria.

### Conjugation assay

To study the ability of evolved multiresistant bacteria to further transfer their resistance-conferring plasmid through conjugation, random individual bacterial clones (both from phage-containing and from phage-free treatments) were transferred from the kanamycin–rifampicin plates to 5 mL of fresh LB medium and then grown overnight at 37°C and 200 rpm. Similar culture was made of strain JM109(pSU19). Next day, the cultures of the multiresistant clones were mixed in 1:1 ratio with JM109(pSU19), and fresh LB medium was added (12.5% of the combined volume of the two bacteria). These cultures were then grown for 24 hours at 37°C without shaking. A sample from each culture was plated on 1% LB agar plate containing chloramphenicol, kanamycin, ampicillin and tetracycline to see whether the RP4 plasmid had transferred itself to JM109(pSU19) and again formed a new multiresistant strain: JM109(pSU19)(RP4). Clones were scored conjugation defective if no colonies formed on chloramphenicol–kanamycin–tetracycline–ampicillin plates. Five randomly selected clones that turned out to be conjugation deficient were further grown in LB medium with JM109(pSU19), now in the presence of chloramphenicol and kanamycin in nonlethal but growth-reducing concentrations (in final concentrations of 0.625 μg/mL and 1.25 μg/mL, respectively (Fig S1C–D)), to see whether the selective pressure posed by the antibiotics would revert the conjugation ability. Five clones that had been capable of conjugation in the first experiment were used as a control. This experiment lasted for 72 hours with the initiation, culture renewing, sampling and plating carried out similarly to the main experiment with the exception of using different antibiotics. The number of potential JM109(pSU19)(RP4) bacteria was measured every day.

### Data analysis

The frequencies of multiresistant bacteria in different treatments were calculated by dividing the density of multiresistant bacteria by the total bacterial density. For statistical tests, arcsine transformation was performed on the obtained frequencies, and the transformed frequencies were compared between phage-free and phage-containing treatments using one-way anova. The level of statistical significance was adjusted with Bonferroni correction to control the effects of multiple comparisons.

## Results

**Figure 2:**
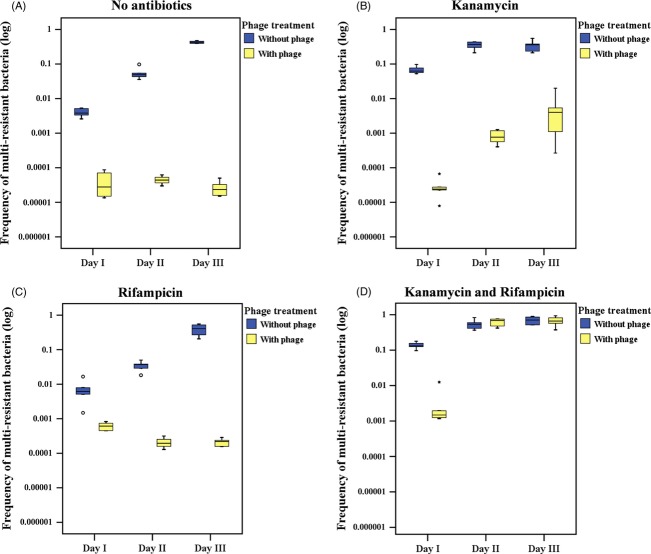
Frequencies of multiresistant (kanamycin + rifampicin) bacteria in the presence and absence of phages. In antibiotic treatments (A) ‘no antibiotics’, (B) ‘kanamycin’ and (C) ‘rifampicin’, there were significantly less multiresistant bacteria in phage-containing treatments throughout the experiment; in treatment (D) ‘kanamycin and rifampicin’, the difference was significant only after the first experimental day (Table S1).

The presence of the conjugative plasmid–dependent phage PRD1 significantly reduced the formation of multidrug-resistant *E. coli* HMS714(RP4) bacteria by infecting all bacteria harbouring actively conjugating resistance–conferring plasmids (Fig. 2). In the absence of phages, multiresistant bacteria quickly became common in both antibiotic-free and all antibiotic-containing treatments. The addition of phages resulted in several orders of magnitude lower levels of multiresistant bacteria. In treatments where neither or only one of the two antibiotics (kanamycin or rifampicin) was present in nonlethal but growth-reducing concentration, the phages reduced the prevalence of multiresistance for the entire length of the three-day experiment (Fig. 2A–C). With the double-antibiotic selection, there were still significantly fewer multiresistant bacteria in the phage-containing treatment after the first experimental day, but by the second day, the difference to the phage-free treatment had mostly disappeared (Fig. 2D). Descriptive statistics for the frequencies of multiresistant bacteria in different treatments and the statistical comparisons thereof (one-way anova) are given in Table S1. The addition of phages did not considerably affect the total number of bacteria (Table S2) in any treatment (all cultivations grew to a saturated density of approximately 10^8^ colony-forming units per millilitre; cfu/mL), but rather had an effect on the relative numbers of different bacterial types in a population, often selecting against the multiresistant ones. Furthermore, infective PRD1 particles were still abundant at the end of the experiment in all phage-containing treatments (Table S3).

Given that multiresistant bacteria were capable of taking over the double-antibiotic system (Fig. 2D) despite the presence of phages, we decided to further investigate the properties of these particular bacteria. They may be harbouring RP4 plasmids with mutations in the cell surface complex that PRD1 uses as a receptor and which is also needed for successful conjugation (Kornstein et al. 1992; Kotilainen et al. 1993). The kanamycin- and rifampicin-resistant clones in all experiments were resistant also to ampicillin and tetracycline, confirming that it was the plasmid and not spontaneous resistance that produced the observed resistance pattern. Nevertheless, such mutated plasmids would provide the host bacteria with simultaneous antibiotic and phage resistance but, due to disturbed conjugation machinery, be unable to transfer the plasmid. Following this line of reasoning, we tested whether the multiresistant bacteria emerged under the simultaneous double-antibiotic and phage selection were still capable of conjugation and, indeed, found that only 35% of randomly selected kanamycin–rifampicin-resistant clones (*n* = 72) were capable of transferring the RP4 plasmid to a third chloramphenicol-resistant *E. coli* strain, JM109(pSU19). Moreover, in three of the total five independent selection experiments, the lost conjugation capability did not revert even after 3 days of subsequent cultivation under antibiotic selection that would have favoured the reversion. In two selection experiments, few multiresistant clones of total ∼5 × 10^8^ bacteria appeared, but they remained at very low quantities (∼10^2^) throughout the three-day experiment. This suggests that after phage exposure, a prolonged selective condition would be required for the potential conjugative multiresistant strains to become abundant in the population. In contrast, all multiresistant bacterial clones isolated from the phage-free treatment retained the conjugation ability.

## Discussion

Our results demonstrate that conjugative plasmid–dependent bacteriophage PRD1 can significantly reduce the horizontal spread of antibiotic resistance genes in a bacterial community even when the bacteria are exposed to antibiotic selection that should favour the evolution of multidrug-resistant strains via conjugation. The addition of conjugative plasmid–dependent phages to any of the antibiotic treatments acted as counter-selection against the spread of multiresistance commonly reducing it by several orders of magnitude. Only the selection specifically for the formation of HMS174(RP4) transconjugants coupled with 48 hours of evolution was a strong enough selective pressure to cancel the differences between the phage-containing and phage-free treatments. However, most bacteria in this phage-containing treatment had also lost their conjugation ability, whereas all bacteria in the phage-free treatment were still capable of conjugation.

It is known that conjugative plasmids can regulate their rate of transfer in several ways (Gasson and Willetts 1975). More specifically to this study, previous empirical work has shown that the presence of PRD1 can select for plasmid-harbouring bacteria that are phage resistant but conjugation deficient (Kotilainen et al. 1993; Jalasvuori et al. 2011). Theoretical models have suggested that heterogeneity in the rate of transfer is essential for the stable maintenance of conjugative plasmids in bacterial communities when conjugative plasmid–dependent phages are present (Dionisio 2005). From this heterogeneity, it follows that phages may be unlikely to be able to completely eradicate conjugative plasmids from a bacterial community but they can, nevertheless, potentially hinder the further spread of plasmid-mediated antibiotic resistances to other bacterial species that may already possess some other resistances (thus being candidates for new multiresistant agents). In our experiments, the lost conjugation ability of a given multiresistant bacterial clone did not seem to revert easily even when the phage selection was lifted and a three-day antibiotic selection favouring the reversion was added.

Recently Zhang and Buckling (2012) demonstrated that the combined bacteriosidic effect of antibiotic kanamycin and a lytic bacteriophage significantly decreased the rate at which bacteria developed resistance against the antibiotic. Therefore, these studies, along with the present results, suggest that it is reasonable to presume the combination of both plasmid-dependent phages with other lytic phages will induce significant constraints for bacteria to maintain resistances, acquire them horizontally or develop resistances *in situ*. However, Escobar-Páramo et al. (2012) showed that application of antibiotic rifampicin against the host bacteria of a phage decreased the survival of phages in the system and would therefore potentially hinder the efficacy of combined phage and antibiotic treatments. We noticed similar effects when rifampicin alone was used in the system. In these experiments, the phage densities at the end of the three-day serial culture were more than 10-fold lower in comparison with other selection pressures (Table S3). This, nevertheless, is what was expected given that rifampicin selects against the initial plasmid-harbouring bacterium JE2571. Yet, the frequency of multiresistant bacteria in the end of the three-day experiment was relatively high in the presence of rifampicin, suggesting that the lower number of phages eased the selection pressure on the formed HMS174(RP4) transconjugants. However, and in contrast to rifampicin experiments, presence of kanamycin alone or both kanamycin and rifampicin elevated the phage densities above those of antibiotic-free experiments. This was also as predicted as kanamycin selects for the plasmid and thus the hosts of phage PRD1.

The concept of preventing the horizontal transfer of antibiotic resistance genes has been explored by a handful of earlier *in vitro* studies that have successfully used different nonphage molecules, phage coat proteins or replicative nonlytic and lytic conjugative plasmid–dependent phages to interfere with the bacterial conjugation (Novotny et al. 1968; Ou 1973; Fernandez-Lopez et al. 2005; Garcillán-Barcia et al. 2007; Lujan et al. 2007; Jalasvuori et al. 2011; Lin et al. 2011). Our study, to our knowledge, is the first one to demonstrate that lytic conjugative plasmid–dependent phages can, in principle, be effective selective agents against conjugative elements and thus the spread of drug resistances even when the bacteria are under sublethal antibiotic selection favouring the horizontal spread of resistance. Such growth-reducing concentrations of antibiotics have been thought to generate new multiresistant strains (Andersson and Hughes 2012). There are, however, important caveats to keep in mind when assessing these promising results. For example, it is unclear whether the evolutionary trajectories observed in this one particular experimental system are also common in other systems with different sets of bacteria, antibiotics, conjugative plasmids and conjugative plasmid–dependent phages. Also more generally, the relevance of results of *in vitro* experiments to the situation in natural environments is always uncertain.

As it currently seems inevitable that the development of new antibiotics will not be able to keep up with the worldwide emergence of resistance in pathogenic bacteria, it is increasingly important that we come up with alternative and complementary methods of treatment. Phage therapy has traditionally been overlooked by the Western medicine, whereas in Eastern Europe and Soviet Union, it was extensively studied and applied, although not always accordingly to the standards and rigour expected in Western science (Alisky et al. 1998; Chanishvili 2012). The reluctance in the West has largely been due to various technical, financial and safety challenges associated with developing and applying phage therapy. However, the worsening resistance epidemic has led to a revived interest in looking into phages as potential antibacterial agents (Lu and Koeris 2011). We suggest that, along with direct attempts to eliminate pathogenic bacteria via phages, the use of conjugative plasmid–dependent viruses could be one interesting avenue to explore. Characteristics of PRD1-like viruses are particularly promising for the development of phage applications. While phages are usually very host specific infecting only some strains of a given species, PRD1 has an extremely wide host range for a phage and it can exploit plasmids from various incompatibility groups (Olsen et al. 1974). PRD1 can also be produced easily in sufficient quantities (Mesquita et al. 2010) and stored stably over long times (Ackermann et al. 2004). Therefore, it may be possible to develop a wide host range cocktail of phages recognizing a wide variety of conjugation apparatuses and be thus usable in different contexts where antibiotic resistances cause problems. For instance, in hospitals, antibiotics are often administrated both before and after a surgical operation to reduce the risk of complications caused by bacterial infections. The number of postoperative hospitalization days under antibiotic treatment correlates positively with the probability of the emergence of life-threatening multiresistant infections (Schentag et al. 1998). Given that antibiotic resistances rise via horizontal gene transfer in various bacterial groups, including both opportunistic pathogens such as *Actinobacter baumannii* (Joshi et al. 2003) and common nosocomial pathogens like *Escherichia coli*, *Klebsiella pneumoniae* (Harajly et al. 2010) and *Staphylococcus aureus* (Lyon and Skurray 1987; Chang et al. 2003; Weigel et al. 2003), the presence of plasmid-dependent phages could hypothetically give antibiotics and the immune system more time to clear the infection before the emergence of highly resistant strains and also restrict the spread of resistances within the hospital in general. Yet, while this concept appears promising, future research in actual *in vivo* systems that are inevitably much more complex in all respects is essential to evaluate the real potential of plasmid-dependent phages.

## Data archiving statement

Data deposited in the Dryad repository: doi:10.5061/dryad.4194c.

## Supporting Information

**Figure S1.** Bacterial growth curves illustrating the effects of lethal and non-lethal antibiotic selection on bacterial growth.

**Table S1.** Arcsine transformed mean frequencies (± standard deviation) of multi-resistant (kanamycin and rifampicin) bacteria) in different antibiotic treatments divided into experimental days, and statistical comparisons (1-way anova) between phage-containing and phage-free treatments.

**Table S2.** Mean total number (± standard deviation) of bacteria (colony-forming units per milliliter; cfu/mL) in different antibiotic treatments divided into experimental days.

**Table S3.** Mean number (± standard deviation) of PRD1 phage particles (particle-forming units per millilitre; pfu/mL) present in different antibiotic treatments at the end of the experiment.
