# Supplementary material for: Fight evolution with evolution: plasmid-dependent phages with a wide host range prevent the spread of antibiotic resistance
Source: Evol Appl. 2013 Jun 10;6(6):925–32. doi: 10.1111/eva.12076 (PMC3779093; doi:10.1111/eva.12076)
Supplement: Supplementary file 1 [file eva0006-0925-SD1.docx]

**Supplementary materials**

**
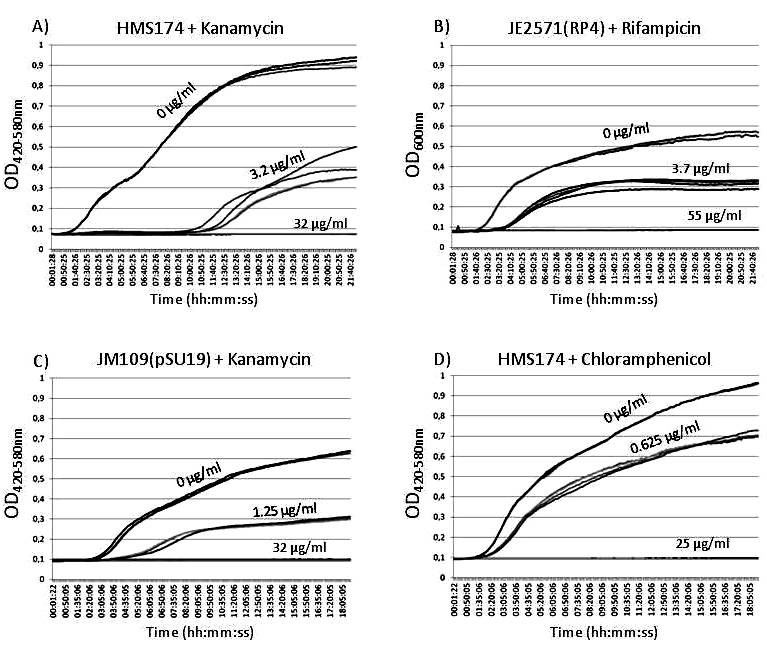
**

**Figure S1** Bacterial growth curves illustrating the effects of lethal and non-lethal antibiotic selection on bacterial growth. 10 μl of overnight cultures (in LB-medium) of E. coli K-12 strains HMS174, JE2571(RP4) and JM109(pSU19) were inoculated into the wells of “Honeycomb 2” plates (Oy Growth Curves Ab Ltd) containing fresh LB-medium and either: 1) no antibiotics, 2) antibiotics in lethal concentration, 3) antibiotics in presumably non-lethal but growth-reducing concentration. Each “bacterium + antibiotic” combination (A-D) had three replicates for treatments 1) and 2), and four replicates for treatment 3). Growth curves were obtained by measuring the optical densities (OD) of the cultures with Bioscreen C spectrophotometer (Oy Growth Curves Ab Ltd). Measurements were done overnight in +37 ºC with no shaking and using either a 420-580 nm wideband filter or a 600 nm brown filter. (A) The effects of lethal (32 μg/ml) and non-lethal (3.2 μg/ml) kanamycin selection on the growth of HMS174. (B) The effects of lethal (55 μg/ml) and non-lethal (3.7 μg/ml) rifampicin selection on the growth of JE2571(RP4). (C) The effects of lethal (32 μg/ml) and non-lethal (1.25 μg/ml) kanamycin selection on the growth of JM109(pSU19). (D) The effects of lethal (25 μg/ml) and non-lethal (0.625 µg/ml) chloramphenicol selection on the growth of HMS174.

**Table S2** Arcsine transformed mean frequencies (±standard deviation) of multi-resistant (kanamycin and rifampicin) bacteria) in different antibiotic treatments divided into experimental days, and statistical comparisons (1-way ANOVA) between phage-containing and phage-free treatments. Each treatment consists of six replicates. Following a Bonferroni correction, the differences between phage-free and phage-containing treatments are considered statistically significant when *p* < 0.01667.

| Antibiotic treatment | Day | Phage treatment | Mean±SD (arsin_freq.) | *F*_1, 10_ | *p*-value |
| --- | --- | --- | --- | --- | --- |
| No antibiotics | I | Without phage | 0.0040±0.0011 | 84.86 | < 0.001 |
|  |  | With phage | 0.000040±0.000031 |  |  |
|  | II | Without phage | 0.055±0.022 | 37.64 | < 0.001 |
|  |  | With phage | 0.000045±0.000012 |  |  |
|  | III | Without phage | 0.44±0.036 | 896.34 | < 0.001 |
|  |  | With phage | 0.000027±0.000013 |  |  |
| Kanamycin | I | Without phage | 0.068±0.017 | 99.12 | < 0.001 |
|  |  | With phage | 0.000029±0.000020 |  |  |
|  | II | Without phage | 0.37±0.10 | 80.76 | < 0.001 |
|  |  | With phage | 0.00083±0.00034 |  |  |
|  | III | Without phage | 0.36±0.13 | 41.56 | < 0.001 |
|  |  | With phage | 0.0058±0.0072 |  |  |
| Rifampicin | I | Without phage | 0.0072±0.0051 | 10.29 | 0.009 |
|  |  | With phage | 0.00060±0.00015 |  |  |
|  | II | Without phage | 0.0349±0.011 | 64.10 | < 0.001 |
|  |  | With phage | 0.00020±0.000070 |  |  |
|  | III | Without phage | 0.4132±0.16105 | 39.46 | < 0.001 |
|  |  | With phage | 0.00021±0.000050 |  |  |
| Kanamycin and Rifampicin | I | Without phage | 0.1374±0.028 | 131.35 | < 0.001 |
|  |  | With phage | 0.0033±0.0045 |  |  |
|  | II | Without phage | 0.59±0.21 | 0.77 | 0.401 |
|  |  | With phage | 0.69±0.19 |  |  |
|  | III | Without phage | 0.80±0.24 | 0.05 | 0.828 |
|  |  | With phage | 0.77±0.28 |  |  |

**Table S3** Mean total number (±standard deviation) of bacteria (colony-forming units per milliliter; cfu/ml) in different antibiotic treatments divided into experimental days. Each treatment consists of six replicates.

| Antibiotic treatment | Day | Phage treatment | Mean±SD (cfu/ml) |
| --- | --- | --- | --- |
| No antibiotics | I | Without phage | 3.77e8±8.73e7 |
|  |  | With phage | 2.82e8±9.50e7 |
|  | II | Without phage | 6.30e8±1.50e8 |
|  |  | With phage | 5.98e8±6.65e7 |
|  | III | Without phage | 4.42e8±1.21e8 |
|  |  | With phage | 5.18e8±1.21e8 |
| Kanamycin | I | Without phage | 4.00e8±5.18e7 |
|  |  | With phage | 2.92e8±6.02e7 |
|  | II | Without phage | 6.47e8±1.94e8 |
|  |  | With phage | 2.47e8±6.86e7 |
|  | III | Without phage | 2.62e8±5.74e7 |
|  |  | With phage | 1.40e8±6.75e7 |
| Rifampicin | I | Without phage | 4.13e8±7.28e7 |
|  |  | With phage | 3.78e8±5.78e7 |
|  | II | Without phage | 5.85e8±1.33e8 |
|  |  | With phage | 5.07e8±8.26e7 |
|  | III | Without phage | 3.42e8±1.25e8 |
|  |  | With phage | 4.53e8±9.37e7 |
| Kanamycin and Rifampicin | I | Without phage | 3.03e8±4.03e7 |
|  |  | With phage | 3.10e8±7.56e7 |
|  | II | Without phage | 6.02e8±1.30e8 |
|  |  | With phage | 3.18e8±1.14e8 |
|  | III | Without phage | 5.80e8±5.83e7 |
|  |  | With phage | 5.10e8±1.39e8 |

**Table S4** Mean number (±standard deviation) of PRD1 phage particles (particle-forming units per millilitre; pfu/ml) present in different antibiotic treatments at the end of the experiment. Each treatment consists of six replicates.

| Antibiotic treatment | Mean±SD (pfu/ml) |
| --- | --- |
| No antibiotics | 7.08e7±2.82e7 |
| Kanamycin | 2.16e9±1.57e9 |
| Rifampicin | 2.83e7±7.53e6 |
| Kanamycin and Rifampicin | 2.23e8±2.10e8 |
